# Using Electronic Health Records to Support Clinical Trials: A Report on Stakeholder Engagement for EHR4CR

**DOI:** 10.1155/2015/707891

**Published:** 2015-10-11

**Authors:** Colin McCowan, Elizabeth Thomson, Cezary A. Szmigielski, Dipak Kalra, Frank M. Sullivan, Hans-Ulrich Prokosch, Martin Dugas, Ian Ford

**Affiliations:** ^1^Robertson Centre for Biostatistics, Institute of Health and Wellbeing, University of Glasgow, Glasgow G12 8QQ, UK; ^2^Department of Internal Medicine, Hypertension and Vascular Diseases, The Medical University of Warsaw, Central Teaching Hospital SP CSK, 1A Banacha Street, 02 097 Warsaw, Poland; ^3^The European Institute for Health Records (EuroRec), 9830 Sint-Martens-Latem, Belgium; ^4^UTOPIAN, University of Toronto, North York General Hospital, 4001 Leslie Street, Room GS-70, Toronto, Canada ON M2K 1E1; ^5^Department of Medical Informatics, Friedrich-Alexander University Erlangen-Nürnberg, 91058 Erlangen, Germany; ^6^Institute of Medical Informatics, University of Münster, 48149 Münster, Germany

## Abstract

*Background*. The conduct of clinical trials is increasingly challenging due to greater complexity and governance requirements as well as difficulties with recruitment and retention. Electronic Health Records for Clinical Research (EHR4CR) aims at improving the conduct of trials by using existing routinely collected data, but little is known about stakeholder views on data availability, information governance, and acceptable working practices.* Methods*. Senior figures in healthcare organisations across Europe were provided with a description of the project and structured interviews were subsequently conducted to elicit their views.* Results*. 37 structured interviewees in Germany, UK, Switzerland, and France indicated strong support for the proposed EHR4CR platform. All interviewees reported that using the platform for assessing feasibility would enhance the conduct of clinical trials and the majority also felt it would reduce workloads. Interviewees felt the platform could enhance trial recruitment and adverse event reporting but also felt it could raise either ethical or information governance concerns in their country.* Conclusions*. There was clear support for EHR4CR and a belief that it could reduce workloads and improve the conduct and quality of trials. However data security, privacy, and information governance issues would need to be carefully managed in the development of the platform.

## 1. Background

Electronic Health Records for Clinical Research (EHR4CR) is a large private-public partnership project which involves 34 academic and private partners, including 11 academic health provider sites across France, Germany, Poland, Switzerland, and the United Kingdom [1]. The project aims to design and build a robust and scalable platform to reuse data from Electronic Health Record (EHR) systems whilst adhering to ethical, regulatory, and data governance policies within each of the countries of the participating sites.

The aim of the EHR4CR platform is to support the conduct of clinical trials, specifically to (1) assist in the assessment of trial feasibility, (2) aid patient recruitment, (3) allow automated preloading of clinical information from a patient's EHR to a trial data collection form, and (4) use EHR information in the reporting of Serious Adverse Events (SAE) during a trial.

There are increasing challenges in the clinical trials environment from economic pressures and regulatory demands. The pharmaceutical industry in particular is investing more in clinical research, year-on-year, but bringing fewer new drugs to the market. Around 57% of pharma research costs are spent on the conduct of clinical trials [2], and yet this stage of drug development contains significant avoidable costs, such as protocol amendments due to recruitment delays [3] (costing around $0.5 million per amendment). However, there is also a growing consensus that the use of EHRs can provide new and unique opportunities to develop more efficient trial processes. Previous work has shown that use of EHRs can improve quality assessment, epidemiological research, and clinical trials within primary care [4], and using EHRs can improve patient recruitment rates to trials [4–6]. However there are still few examples of where research data are integrated with patient clinical data [7] and there are concerns around meeting privacy, regulatory, and data governance requirements when using EHRs for research [8, 9].

To better understand how different technical approaches would be seen in terms of ethical, regulatory, and data governance attitudes across the different countries we decided to conduct a series of structured interviews with senior figures in healthcare organisations across Europe (including managers and information governance staff, academics, clinical opinion leaders, ethicists, and research funders).

## 2. Methods

Materials for the conduct of structured interviews with senior figures in healthcare organisations across Europe (including managers and information governance staff, academics, clinical opinion leaders, ethicists, and research funders) were developed based on detailed interviews conducted in a pilot study in Glasgow, Scotland [10]. The final interview schedule consisted of a series of questions relating to different aspects of the project which the interviewee responded by using a five-point Likert scale (Strongly Agree/Agree/Neither/Disagree/Strongly Disagree) and a number of free text responses for other issues not raised within the set questions.

The principal investigator at each of the pilot sites participating in EHR4CR was contacted and asked to conduct interviews for the stakeholder engagement survey with key people in their own geographic area using a snowball sampling technique [11]. The selection of participants was left to the discretion of each site so they could best utilise their local knowledge and networks, although different categories of interviewee were defined. Potential interviewees were contacted and asked to participate and if they agreed a time was set either for a face-to-face or telephone interview, dependent on the interviewees' preferences. The interviews were conducted by senior members of project staff (usually the PI) in the second half of 2012 and the first half of 2013.

The interviewees were provided with a description of the project and structured interviews were conducted initially focusing on the four project objectives (trial feasibility, facilitating recruitment, facilitating clinical trial delivery, and reporting of adverse drug events). Within each objective a series of scenarios as illustrated below were presented and related questions posed.

## 3. Trial Feasibility

Access to patient data is required to inform trial design and trial feasibility assessment and so information and governance approval would be required for the generic process rather than a specific trial. A clinical research sponsor would be interested in examining the prevalence or incidence of a disease or the rate of clinical outcomes of interest. By comparing a defined set of inclusion/exclusion criteria against data held at a number of centres this would help improve trial design, in particular by estimating the likely number of patients who would match the eligibility criteria, thereby allowing a more accurate prediction of recruitment rates per site.

The first part of the interview questionnaire presented four different options, as Scenarios A–D, for the kind of information that could be extracted from a hospital EHR system and returned to the research sponsor.

*Scenario A*. Only the total number or percentage of eligible patients meeting all criteria is returned to the research sponsor.

*Scenario B*. The total number or percentage of eligible patients meeting each individual criterion is returned to the research sponsor.

*Scenario C*. Total number or percentage of eligible patients meeting each criterion is returned to a third party to work on behalf of the research sponsor.

*Scenario D*. Deidentified individual patient records relating to the eligibility criteria are returned to the research sponsor.

A set of questions were posed regarding the acceptability of each of the four scenarios, from different ethical and governance perspectives.

## 4. Facilitating Recruitment

Once a clinical trial protocol has been finalised, the EHR4CR platform could transmit the patient eligibility criteria electronically to each participating hospital or another participating organisation, for example, community mental health team. This part of the interview questionnaire explored the acceptability of using these electronic criteria to support a hospital to identify and contact potentially eligible patients (Scenario E).

*Scenario E*. Routinely collected patient data could be used to facilitate the identification of potentially eligible recruits for the trial at a centre, given that all relevant permissions are in place. The study inclusion/exclusion criteria would be provided and run on behalf of the investigator against the local database to extract a list of potentially eligible patients. The local investigator could select individuals from the list as appropriate and generate letters of invitation to participate in the trial to the patients. No individual patient level data would be returned to the organisation conducting the clinical research prior to patient consent.

## 5. Facilitating Clinical Trial Execution

The next two scenarios related to information on patients who have been recruited into the trial and provided full informed consent for the use of their electronic health record.

*Scenario F*. Data is extracted from the electronic health record into the trial database to facilitate trial conduct. There would be an option to allow the investigator to approve each data transfer.

*Scenario G*. Data collected specifically for the trial is added to the patient's electronic health record.

Interviewees were asked to comment on the acceptability of each of these two scenarios, which are not mutually exclusive.

## 6. Reporting of Adverse Drug Events

The final four scenarios focused on the electronic extraction and communication of data about a serious adverse event occurring during a clinical trial. These scenarios portrayed different aggregation levels of patient data.

*Scenario H*. Individual patient records relating to adverse event data and associated clinical and prescribing data are returned to the organisation conducting the clinical research.

*Scenario I*. Individual patient records relating to adverse event data and associated clinical and prescribing data are returned to a local clinician to support the completion and submission of each ADE to the marketing authority and the regulatory agency, as appropriate.

*Scenario J*. Automated extraction of periodic aggregated summary information on adverse events extracted from electronic records and reported to the marketing authority and the regulatory agency, as appropriate.

A final scenario (Scenario K) describing the use of an existing national dataset of deidentified patient data was described and interviewee responses to related questions were collected.

## 7. Motivations and Threats

In addition, a series of questions were posed relating to (a) motivating factors for a hospital/academic institution to participate in trials using the proposed EHR4CR platform and (b) threats and challenges to the success of an EHR4CR platform to support trials. Finally, interviewees were asked to give their overall impression of the EHR4CR project.

Responses from each interviewee were recorded using a standardised form relating to the specific questions asked within the interview. These were then anonymised and returned to the Robertson Centre for Biostatistics, University of Glasgow, where they were entered into a study database. The individual identities of each interviewee were not recorded. Each interviewee could be categorised by country and their job role, although some may have had multiple roles (e.g., clinical and academic). Individual responses to set questions were reported using the frequency for each response category. Free text responses were reviewed but proved not to be very helpful with no additional information taken from these.

## 8. Results

There were a total of 37 structured interviews conducted by the leads at 7 different pilot sites for EHR4CR in Germany, the UK, Switzerland, and France with the breakdown of personnel type interviewed and their location shown in Table 1. The leads at the centres reported conducting a mix of telephone and face-to-face interviews with each lasting between 60 to 90 minutes, although data on format and timing were not collected.

### 8.1. Trial Feasibility

Interviewees were unanimous that an EHR4CR platform approach to assessing feasibility would enhance the conduct of clinical trials and a clear majority of responders indicated that they anticipated that this approach would reduce workloads in the assessment of clinical trial feasibility for clinical and research staff at the participating centres (24% Strongly Agree and 47% Agree).

The majority of respondents reported that prior informed consent would not be required (81%), their institution would approve data transfer (81%), and there would be no ethics/information governance concerns (74%) for the return of a single aggregated number of eligible patients as outlined in Scenario A (see Table 2). Scenario B, which suggested returning aggregated numbers for each criterion, had slightly lower levels of support: prior informed consent not needed (70%), institution approved (70%), and no ethics/information governance concerns (65%).

The scenarios suggesting the return of anonymous data to a third party (Scenario C) or the research sponsor (Scenario D) had markedly different results. The respondents reported that prior informed consent would be needed (68%), less than half felt their institution would approve data transfer (43%), and there would be ethics/information governance concerns (65%) using a third party to host data. Returning data to the research sponsor would need prior informed consent (78%), less than half felt their institution would approve data transfer (41%), and the vast majority suggested there would be ethics/information governance concerns (85%).

### 8.2. Trial Recruitment

The majority of respondents (70%) thought that using EHR4CR for recruitment to trials would be approved by healthcare organisations in their country and that these approaches would also enhance the conduct of trials (67%) (see Table 3). However, approximately 50% of responders thought that using EHR4CR for recruitment would create either ethics or information governance concerns in their country and would require prior informed consent (51%) and prior regulatory approval (57%).

### 8.3. Facilitating Trial Execution

The respondents reported that using EHR4CR to extract data from the patient's electronic records to the trial dataset would be approved by healthcare organisations in their country (70%) and would not raise ethics/information governance concerns (71%) (see Table 4). There was less support for the transfer of trial data back to the patient's record: only 49% thought it would be approved by their institution, whilst 47% thought it would raise ethics/information governance concerns. Respondents suggested the transfer of trial specific data into electronic health records could lead to some concerns for treating physicians (65%) but this could be reduced by holding this data separately (59%).

### 8.4. Adverse Event Reporting

Using EHR4CR to facilitate adverse event reporting was widely accepted as enhancing trials (see Table 5). Although only 50% were confident that this would receive ethical approval for return of all data to the research sponsor (Scenario H), this increased if data were dealt with by a local clinician (Scenario I, 70%) or to the regulatory authority (Scenario J, 59%). Half (50%) of respondents thought returning adverse event reporting data to the research sponsor would raise ethics/information governance concerns, but this reduced to a quarter (24%) if returned to a local clinician (24%) or a third (32%) if returned to the regulatory authority (32%).

## 9. Motivations and Threats

The strongest motivating factors for future participation in an EHR4CR platform (see Table 6) were income generation from industry trials (Strongly Agree 16% and Agree 54%), providing patients with faster access to novel medicines (Strongly Agree 27% and Agree 46%), stimulus for the development of local health information systems (Strongly Agree 22% and Agree 51%), improved quality of healthcare data (Strongly Agree 38% and Agree 41%), potential to use the platform for academic studies (Strongly Agree 35% and Agree 49%), improvement in the quality (Strongly Agree 32% and Agree 46%), and efficiency (Strongly Agree 30% and Agree 54%) of trials. The biggest threats raised to the success of EHR4CR were considered to be the inadequacy of local health information systems (Strongly Agree 16% and Agree 43%); missing key data items (Strongly Agree 16% and Agree 46%); the cost of upgrading local system environments (Strongly Agree 32% and Agree 27%), and ethical (Strongly Agree 8% and Agree 49%), data protection (Strongly Agree 11% and Agree 46%), and information governance (Strongly Agree 14% and Agree 51%) concerns.

## 10. Discussion

The overwhelming message from stakeholder engagement was the positive support for the proposed EHR4CR platform to enhance the conduct of feasibility assessments and the recruitment of study subjects and thereby to improve the conduct of clinical trials. However respondents highlighted that the platform may raise ethical and governance concerns in all areas and failing to meet these requirements would constitute major threats to the project. The requirement for regulatory and institutional support within the proposed feasibility scenarios suggested strongly that patient data should not be transferred outside the host institution and also that patients should be given an opportunity to opt out of use of their EHR.

Stakeholder support for the project was shown through agreement that it would improve the local quality of care offered to patients, could improve local health systems, and would help patients get access to new medications faster. Increased participation for local centres in trials and additional financial benefits associated with this were also highlighted, although few stakeholders suggested these as reasons to participate.

The EHR4CR stakeholder engagement raised many important issues about governance, privacy, and data management and data standards. The development of the EHR4CR project and how it addressed these barriers and challenges will provide information to influence future developments in big healthcare databases. With the ultimate goal of improving clinical trials in the European Union, the project provides a unique opportunity to coordinate important stakeholders' efforts to create new clinical trials environments.

## 11. Strengths and Limitations of the Work

Although this survey was targeted at informing the design and governance of the EHR4CR platform, the scenarios and the interview questions were posed in a generic form which relates to the use of hospitals electronic health record system to support the design, recruitment, and conduct of clinical trials. Interviewees therefore did not need to have a detailed understanding of the project or its particular technical implementation in order to respond to the survey.

The seniority of the individuals invited for interview and the length of the interview (often between 60 and 70 minutes) will have placed practical limits on the number of participants per country, and the number of countries which could be included in this survey. The interviews were arranged and conducted by senior academics who were aware of the potential risk of bias and each deliberately sought to invite interview senior individuals who were not otherwise connected with the project or with the partner organisations in the consortium but would be important decision makers or decision influences in the wider acceptability of the proposed approach. Nevertheless it is recognised that this survey was by invitation and that this sample cannot be claimed to be fully representative of the stakeholder groups included.

## 12. Comparison to the Literature

There are a number of other initiatives looking to improve clinical research through innovative uses of routine data. The Sentinel Initiative was launched by the FDA in 2008, to develop and implement a proactive system to track the safety of drugs, biologics, and medical devices once they reach the market. It uses preexisting electronic healthcare data at collaborating institutions by running a centrally developed computer program at each site which returns summary results to the organising centre [12]. The system has been used effectively in a number of recent drug safety studies [13–15].

Other initiatives include the Observational Medical Outcomes Partnership (OMOP) [16] and SHRINE [17], which are public-private partnerships built upon the use and sharing of information from existing observational databases. The development of initiatives such as these would suggest that the question is no longer whether but rather how clinical data should be shared to foster innovation and support clinical research [18].

However, it is still unknown how the process of data sharing can become routine, how to define responsible data sharing, which principles to establish, and how to set policies across different countries where interpretations of clinical and information governance may vary and where attitudes towards reuse of routinely collected clinical data may differ. The EHR4CR project may be one of the milestones on the road ahead.

## 13. Conclusions

There was clear support for the overall objectives of EHR4CR and a belief that a well-developed EHR4CR platform would reduce workloads and improve the conduct and quality of trials. However, the interviewees did raise some ethical and information governance concerns and threats to the potential success of an EHR4CR platform including that only aggregated data should be reported for trial feasibility, that prior ethics approval may be required for use of patient's EHR for recruitment, and that the combination of trial data with patient EHR could generate new issues and concerns. This study has helped guide the development of the EHR4CR informatics platform and highlight areas where there is a need to clarify and emphasise data security, privacy, and information governance issues in the roll-out of the platform.

## Supplementary Material

The complete questionnaires used to conduct the interviews are available as Supplementary Materials to this paper online. There were two interview schedules used-one for staff involved with Ethics committees and one for other staff categories.

## Figures and Tables

**Table 1:** Country and job category of respondents.

	Number of respondents *n* = 37
*Country *	
UK	11
Germany	16
Switzerland	8
France	2

*Job Category* ^*∗*^	
Healthcare organisation, general management	3
Healthcare organisation, research management	5
Healthcare organisation, information governance officer	3
Senior clinical researcher/CTU director	7
Senior informatics staff	4
Healthcare service provider	10
National policy makers	3
National opinion leaders	4
Patient association lead	3
Ethics committee representative	3

^*∗*^Participants may be in more than one category.

**Table 2:** Use of the EHR4CR platform for trial feasibility.

	Scenario	A	B	C	D
Do you think that data transfer would require previous informed consent by patients for the use of their data in this manner?	Yes	7 (19)	10 (27)	25 (68)	29 (78)
	No	30 (81)	26 (70)	8 (22)	5 (14)
	Do not know	0	1 (3)	3 (8)	3 (8)

Do you think that data transfer would be approved by your institution (or an institution in your country if you are not based in a healthcare institution)/by an ethics committee in your country?	Yes	30 (81)	26 (70)	16 (43)	12 (32)
	No	3 (8)	5 (14)	9 (24)	15 (41)
	DK	1 (3)	2 (5)	8 (22)	6 (16)

Do you think that the transfer of these data would create ethical/information governance concerns at your institution (or an institution in your country if you are not based in a healthcare institution)?	Yes	7 (21)	7 (21)	22 (65)	29 (85)
	No	25 (74)	22 (65)	7 (21)	2 (6)
	DK	1 (3)	4 (12)	4 (12)	2 (6)

	Strongly Agree	Agree	Neither	Disagree	Strongly Disagree

Indicate your agreement/disagreement with the statement that “this approach to facilitating feasibility assessment would enhance the conduct of clinical trials.”	0	3 (100)	0	0	0

Indicate your agreement/disagreement with the statement that “providing data to an organisation conducting clinical research or trusted third party in such an automated manner would reduce workloads and save time of healthcare institution employees.”	8 (24)	16 (47)	6 (18)	4 (12)	0

Scenario A: total number of patients meeting all criteria only returned to sponsor.

Scenario B: number of patients meeting each criterion returned to sponsor.

Scenario C: number of patients meeting each criterion returned to 3rd party.

Scenario D: deidentified patient records meeting criteria returned to sponsor.

**Table 3:** Use of the EHR4CR platform for trial recruitment (Scenario E).

	Yes	No			DK
Indicate whether you think that this scenario would require previous informed consent by patients for the use of their data by the investigator/by the healthcare team.	19 (51)	16 (41)			1 (3)

Indicate whether you think that this scenario would require previous authorisation from data protection authority or another external regulatory body.	21 (57)	11 (30)			4 (11)

Do you think that this scenario would be accepted by your institution (or an institution in your country if you are not based in a healthcare institution)/do you think, in your opinion, that this scenario would be approved by an ethics committee in your country?	26 (70)	6 (16)			5 (14)

Indicate your agreement/disagreement with the statement that “this approach to facilitating patient recruitment would enhance the conduct of clinical trials.”	Strongly Agree 0	Agree 2 (67)	Neither 1 (33)	Disagree 0	Strongly Disagree 0

Do you think that this scenario would create ethical/information governance concerns at your institution (or an institution in your country if you are not based in a health care institution)?	Yes 17 (50)		No 16 (47)		DK 1 (3)

Indicate your agreement/disagreement with the statement that “this approach to facilitating patient recruitment would reduce workloads and save time of healthcare institution employees.”	Strongly Agree 11 (32)	Agree 18 (53)	Neither 4 (12)	Disagree 0	Strongly Disagree 0

**Table 4:** Use of the EHR4CR platform for trial execution.

	Scenario		F		G
Do you think that this scenario would be accepted by your institution (or an institution in your country if you are not based in a healthcare institution)/do you think that this scenario would be approved by an ethics committee in your country?	Yes No DK		26 (70) 4 (11) 5 (14)		18 (49) 10 (27) 5 (14)

Indicate your agreement/disagreement with the statement that “this approach to facilitating trial conduct would enhance the quality of clinical trials.”	Strongly Agree		0		1 (33)
	Agree		1 (33)		0
	Neither		2 (67)		0
	Disagree		0		2 (67)
	Strongly Disagree		0		0

Do you think that this scenario would create ethical/information governance concerns at your institution (or an institution in your country if you are not based in a healthcare institution)?	Yes No DK		8 (24) 24 (71) 1 (3)		16 (47) 14 (41) 2 (6)

Indicate how much you would support the statement that “extraction of data automatically from the electronic patient record into a trial database would reduce workloads and save time of healthcare institution employees.”	Strongly Agree 19 (56)	Agree 8 (24)	Neither 3 (9)	Disagree 2 (6)	Strongly Disagree 0

	Yes	No			DK

Do you think that this scenario could create concerns that the additional information might be misunderstood by other physicians treating the patient due to unfamiliar measurements or measurements obtained by unfamiliar methods?	24 (65)	9 (24)			1 (3)

Do you think that this scenario would create fewer concerns if the additional information was separated from the usual patient record?	22 (59)	8 (22)			4 (11)

Scenario F: extraction of data from the patient record.

Scenario G: transfer of trial specific data to the patient's electronic record.

**Table 5:** Use of the EHR4CR platform for adverse event reporting.

	Scenario	H	I	J
Do you think that this scenario would be accepted by your institution (or an institution in your country if you are not based in a healthcare institution)?/do you think that this scenario would be approved by an ethics committee in your country?	Yes No DK	19 (51) 9 (24) 7 (19)	26 (70) 4 (11) 5 (14)	22 (59) 4 (11) 7 (19)

Do you think that this scenario would create ethical/information governance concerns at your institution (or an institution in your country if you are not based in a health care institution)?	Yes	17 (50)	8 (24)	12 (32)
	No	16 (47)	22 (65)	21 (57)
	DK	0	3 (9)	2 (5)

Indicate your agreement/disagreement with the statement that “accumulating adverse event reports in this manner will significantly improve the reporting of adverse drug reactions during clinical trials”/indicate your agreement/disagreement with the statement that “this approach to facilitating adverse event reporting would enhance the evaluation of the safety of medicines.”	Strongly Agree	7 (19)	15 (41)	8 (22)
	Agree	18 (49)	16 (43)	17 (46)
	Neither	10 (27)	4 (11)	7 (19)
	Disagree	1 (3)	0	4 (11)
	Strongly Disagree	1 (3)	1 (3)	0

Indicate whether you think that this scenario would require previous informed consent by patients for the use of their data.	Yes		17 (46)	12 (32)
	No		15 (41)	21 (57)
	DK		3 (8)	2 (5)

Scenario H: individual patient level data on adverse events returned to organisation conducting research.

Scenario I: individual patient level data on adverse events returned to local clinician to prepare report for regulatory authorities.

Scenario J: periodic aggregated data on adverse events reported turned to regulatory authorities.

**Table 6:** Motivators and threats for participation in EHR4CR.

Use of existing national datasets of deidentified data					
Do you think that out-licensing from your institution (or from an institution in your country if you are not based in a healthcare institution) of a large body of detailed pseudoanonymised longitudinal secondary care (hospital) data to an organisation conducting research into postmarketing drug safety would:	Yes		No	Do not know	
(a) Require prior patient level consent?	22 (59)		14 (38)	0	
(b) Be likely to receive institutional approval?	22 (59)		6 (16)	8 (22)	
(c) Raise significant ethical/information security concerns?	21 (57)		14 (38)	1 (3)	
(d) Require data protection authority or another regulatory external body approval?	26 (70)		5 (14)	4 (11)	

Other aspects					

Indicate your agreement/disagreement with the following as strong motivating factors for your institution's participation in the EHR4CR platform (now or in the future). If you are not based in a healthcare institution, consider these factors for an institution in your country.	Strongly Agree	Agree	Neither	Disagree	Strongly Disagree
Increased income generation from participation in more industry trials	6 (16)	20 (54)	1 (3)	5 (14)	4 (11)
Pressure from government or institution to participate in more pharma industry studies	6 (16)	8 (22)	8 (22)	11 (30)	3 (8)
Providing patients with faster access to new generation medicines	10 (27)	17 (46)	4 (11)	3 (8)	2 (5)
Development of local health information systems	8 (22)	19 (51)	6 (16)	1 (3)	2 (5)
Improvement of local data quality and healthcare	14 (38)	15 (41)	3 (8)	3 (8)	1 (3)
The potential to use EHR4CR platform to conduct academic studies	13 (35)	18 (49)	3 (8)	1 (3)	0
Opportunity to improve the quality of data in clinical trials	12 (32)	17 (46)	3 (8)	3 (8)	1 (3)
Opportunity to improve the efficiency of clinical trials	11 (30)	20 (54)	5 (14)	0	0

Indicate your agreement/disagreement with the following as significant threats in your institution or country to the success of EHR4CR.					

Inadequate availability of key data fields in the patient record	6 (16)	16 (43)	6 (16)	6 (16)	3 (8)
Missing data in the patient record	6 (16)	17 (46)	7 (19)	7 (19)	0
Inadequacy of local health information systems	6 (16)	16 (43)	2 (5)	10 (27)	2 (5)
Cost of upgrading local systems to be compatible with EHR4CR	12 (32)	10 (27)	9 (24)	6 (16)	0
Ethical committee concerns	3 (8)	18 (49)	4 (11)	11 (30)	1 (3)
Local information governance concerns	5 (14)	19 (51)	4 (11)	9 (24)	0
Data protection authorities	4 (11)	17 (46)	5 (14)	7 (19)	2 (5)
Concerns of hospital management	2 (5)	13 (35)	8 (22)	13 (35)	1 (3)
Concerns of patients	3 (8)	11 (30)	5 (14)	16 (43)	2 (5)
Concerns of clinicians	0	13 (35)	5 (14)	16 (43)	3 (8)
