# Supplementary material for: Using Electronic Health Records to Support Clinical Trials: A Report on Stakeholder Engagement for EHR4CR
Source: Biomed Res Int. 2015 Oct 11;2015:707891. doi: 10.1155/2015/707891 (PMC4619877; doi:10.1155/2015/707891)
Supplement: Supplementary file 1 — The complete questionnaires used to conduct the interviews are available as Supplementary Materials to this paper online. There were two interview schedules used-one for staff involved with Ethics committees and one for other staff categories. [file 707891.f1.docx]

**
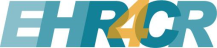
 Stakeholder interaction; non - Ethics**

EHR4CR Partner (interviewer)_______________________________ (*select from list*)

Interviewer Details:

| **Interviewer (*PRINT NAME*):** |  |
| --- | --- |
| **Interview Date:** |  |
| **Interviewer Email address:** |  |

By ticking this box you are giving us permission to store the above data for the purposes of reporting to the EHR4CR project team. All data will be subject to the Data Protection Act 1998

Interviewee category (select from list)

| - Healthcare Organisation - General Management |  |
| --- | --- |
| - Healthcare Organisation - Research Management |  |
| - Healthcare Organisation - Information Governance Officer |  |
| - Senior Clinical Researcher/ CTU Director |  |
| - Senior Informatics Staff |  |
| - Healthcare Service Provider |  |
| - Data Access Regulatory Body |  |
| - National policy makers |  |
| - National opinion leaders |  |

**
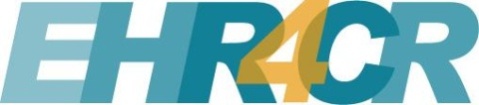
**

**Stakeholder interaction – Non - Ethics**

**Project Areas A - D**

**(Questions 1 – 27)**

**
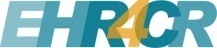
 Project Area A:**

**Using the EHR4CR Platform for Assessing Clinical Trial Feasibility**

**Background**

In this context, access to patient data would be required to inform trial design and trial feasibility assessment. Therefore, ethical and information governance approval would be required for a generic process not necessarily associated with any particular trial.

*Informing study design:* The development of a trial design by an organisation involved in clinical research might require basic knowledge about the number of prevalent cases or the throughput of incident cases with a particular condition. Alternatively, there might be a need to know about the biological variability in a measurement of interest or in the rate of clinical outcomes of interest (e.g. death, re-admission etc).

*Informing feasibility:* Once a protocol has been outlined or even finalised, there will be a need to assess the number of potentially eligible patients at each clinical site to assess the feasibility of the study and to inform the required number of sites and the best sites to participate in the trial.

Infrastructure to answer the questions posed above can be established in different ways.

**Scenario A;** An electronic query would be sent to each participating centre that would be run against a local database established for clinical research purposes returning summary information only (e.g. counts and percentages). Data will be returned only if counts are sufficiently large to protect privacy. For example, information might be returned on the percentage of patients eligible for inclusion in a study.

**Scenario B;** An electronic query would be sent to each participating centre that would require the return of summary information cross-tabulated by a number of key inclusion/exclusion criteria. Data will be returned only if counts are sufficiently large to protect privacy. For instance, the number of eligible participants might be returned for combinations of gender (male/female) and diabetes status (not diabetic/type I/type II).

**Scenario C;** An electronic query would be sent to each participating centre that would require the return of pseudo-anonymised individual patient records containing patient level information on key inclusion/exclusion criteria and other variables of interest. On this occasion, the data would be collated at a trusted third party site, in Europe, so that the impact of variations in study design criteria could be explored. The records would not contain any patient identifiers; date of birth would be converted into age and recorded to nearest year.

**Scenario D;** An electronic query would be sent to each participating centre that would require the return of de-identified individual patient records containing patient level information on key inclusion/exclusion criteria. On this occasion, the data would be returned to the organisation conducting the clinical research that initiated the query, so that the impact of variations in study design criteria could be explored.

**Question 1**

For **each scenario**, indicate whether you think that data transfer would require previous informed consent by patients for the use of their data in this manner.

(Tick one box for each scenario)

| Scenario A | Yes |  |  | Don’t know |  |
| --- | --- | --- | --- | --- | --- |
|  | No |  |  | Not applicable |  |
|  |  |  |  |  |  |
| Scenario B | Yes |  |  | Don’t know |  |
|  | No |  |  | Not applicable |  |
|  |  |  |  |  |  |
| Scenario C | Yes |  |  | Don’t know |  |
|  | No |  |  | Not applicable |  |
|  |  |  |  |  |  |
| Scenario D | Yes |  |  | Don’t know |  |
|  | No |  |  | Not applicable |  |

Provide any additional comments you might have on this topic;

**Question 2**

For **each scenario**, indicate whether you think that data transfer would be approved by your institution (or an institution in your country if you are not based in a health care institution).

(Tick one box for each scenario)

| Scenario A | Yes |  |  | Don’t know |  |
| --- | --- | --- | --- | --- | --- |
|  | No |  |  | Not applicable |  |
|  |  |  |  |  |  |
| Scenario B | Yes |  |  | Don’t know |  |
|  | No |  |  | Not applicable |  |
|  |  |  |  |  |  |
| Scenario C | Yes |  |  | Don’t know |  |
|  | No |  |  | Not applicable |  |
|  |  |  |  |  |  |
| Scenario D | Yes |  |  | Don’t know |  |
|  | No |  |  | Not applicable |  |

Provide any additional comments you might have on this topic

**Question 3**

For **each scenario**, indicate whether you think, that the transfer of these data would create ethical/ information governance concerns at your institution (or an institution in your country if you are not based in a health care institution).

(Tick one box for each scenario)

| Scenario A | Yes |  |  | Don’t know |  |
| --- | --- | --- | --- | --- | --- |
|  | No |  |  | Not applicable |  |
|  |  |  |  |  |  |
| Scenario B | Yes |  |  | Don’t know |  |
|  | No |  |  | Not applicable |  |
|  |  |  |  |  |  |
| Scenario C | Yes |  |  | Don’t know |  |
|  | No |  |  | Not applicable |  |
|  |  |  |  |  |  |
| Scenario D | Yes |  |  | Don’t know |  |
|  | No |  |  | Not applicable |  |

**Question 4**

Indicate your agreement/disagreement with the statement that 'Providing data to an organisation conducting clinical research or trusted third party in such an automated manner would reduce workloads and save time of healthcare institution employees.'

(Tick one box)

Strongly agree Agree Neither Disagree Strongly disagree

Provide any additional comments you might have on this topic

**
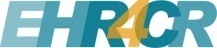
 Project Area B:**

**Use of the EHR4CR Platform for Facilitating Recruitment to Trials**

**Background**

**Scenario E;** Once a trial design has been finalised, all clinical trial approvals obtained and clinical investigators recruited and contracts completed, there is the opportunity to use routinely collected patient data to facilitate the identification of potentially eligible recruits for the trial. In this context the study inclusion/exclusion criteria would be provided in electronic form to the clinical site. An application would be run on behalf of the investigator against the local database to extract a list of potentially eligible patients for the local investigator to scrutinise, delete individuals from the list as appropriate based on local knowledge, and where appropriate generate letters of invitation to participate in the trial to the patients. No individual patient level data would be returned to the organisation conducting the clinical research prior to patient consent.

**Question 5a**

Indicate whether you think that this scenario would require previous informed consent by patients for the use of their data by the investigator/ by the healthcare team.

(Tick one box)

|  | Yes |  |  | Don’t know |  |
| --- | --- | --- | --- | --- | --- |
|  | No |  |  | Not applicable |  |

**Question 5b**

Indicate whether you think that this scenario would require previous authorisation from Data Protection Authority or other external regulatory body.

(Tick one box)

|  | Yes |  |  | Don’t know |  |
| --- | --- | --- | --- | --- | --- |
|  | No |  |  | Not applicable |  |

**Question 6**

Do you think that this scenario would be accepted by your institution (or an institution in your country if you are not based in a health care institution)?

(Tick one box)

|  | Yes |  |  | Don’t know |  |
| --- | --- | --- | --- | --- | --- |
|  | No |  |  | Not applicable |  |

**Question 7**

Do you think that this scenario would create ethical/ information governance concerns at your institution (or an institution in your country if you are not based in a health care institution)?

(Tick one box)

|  | Yes |  |  | Don’t know |  |
| --- | --- | --- | --- | --- | --- |
|  | No |  |  | Not applicable |  |

**Question 8**

Indicate your agreement/disagreement with the statement that 'This approach to facilitating patient recruitment would reduce workloads and save time of healthcare institution employees'.

(Tick one box)

Strongly agree Agree Neither Disagree Strongly disagree

Provide any additional comments you might have on this topic:

**
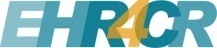
 Project Area C:**

**Use of the EHR4CR Platform for Facilitating Clinical Trial Delivery**

**Background**

In this context, it is assumed that the patient has been recruited into the trial and provided full informed consent for the extraction of data from their electronic record and for addition of new information into the patient record.

**Scenario F;** Extraction of data from the patient record: Accurate collection of clinical trial data can be time consuming, often requiring manual transcribing of information already held in patient routinely collected health records and resolution of queries caused by inaccurate data entry. Examples of data that might be available in electronic records are, demographics, medical history, prescription information, routine measurements (e.g. Blood Pressure, Body Mass Index, pulse). The ability to upload high quality data automatically into the trial database could facilitate trial conduct. There would be an option to allow the investigator to approve each data transfer.

**Question 9**

Do you think that that this scenario would be approved by your institution (or an institution in your country if you are not based in a health care institution)?

(Tick one box)

|  | Yes |  |  | Don’t know |  |
| --- | --- | --- | --- | --- | --- |
|  | No |  |  | Not applicable |  |

**Question 10**

Do you think that this scenario would create ethical/ information governance concerns at your institution (or an institution in your country if you are not based in a health care institution)?

(Tick one box)

|  | Yes |  |  | Don’t know |  |
| --- | --- | --- | --- | --- | --- |
|  | No |  |  | Not applicable |  |

**Question 11**

Indicate how much you would support the statement that 'Extraction of data automatically from the electronic patient record into a trial database would reduce workloads and save time of healthcare institution employees'.

(Tick one box)

Strongly agree Agree Neither Disagree Strongly disagree

Provide any additional comments you might have on this topic:

**Scenario G;** Return of trial specific data to the patient’s electronic record: Data collected for trial purposes that supplement what would be collected in clinical practice are seldom returned to the patient’s record. The ability to automatically enter such information into the patient’s record could in theory enhance future patient care. However, some have raised concerns that data collected out of the usual clinical context, using non-standard techniques and possibly including measurements with unproven clinical relevance, could cause confusion and might not always be helpful for clinical management. Such information could arise from more frequently recorded measurements, specialised measurements on the patient or from images, ECGs, laboratory or tissue samples, PRO, QoL data etc.

**Question 12**

Do you think that this scenario would be accepted by your institution (or an institution in your country if you are not based in a health care institution)?

(Tick one box)

|  | Yes |  |  | Don’t know |  |
| --- | --- | --- | --- | --- | --- |
|  | No |  |  | Not applicable |  |

**Question 13**

Do you think that this scenario would create ethical/ information governance concerns at you institution (or an institution in your country if you are not based in a health care institution)?

(Tick one box)

|  | Yes |  |  | Don’t know |  |
| --- | --- | --- | --- | --- | --- |
|  | No |  |  | Not applicable |  |

**Question 14**

Do you think that this scenario could create concerns that the additional information might be misunderstood by other physicians treating the patient due to unfamiliar measurements or measurements obtained by unfamiliar methods?

(Tick one box)

|  | Yes |  |  | Don’t know |  |
| --- | --- | --- | --- | --- | --- |
|  | No |  |  | Not applicable |  |

**Question 15**

Do you think that this scenario would create fewer concerns if the additional information was separated from the usual patient record?

(Tick one box)

|  | Yes |  |  | Don’t know |  |
| --- | --- | --- | --- | --- | --- |
|  | No |  |  | Not applicable |  |

Provide any additional comments you might have on this topic, highlighting any benefits you might see in this approach:

**
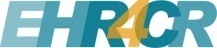
 Project Area D**

**Using the EHR4CR Platform for Adverse Event Reporting**

**Background**

An adverse event is a change to a patient’s health occurring after initiation of a treatment. Collection of data on adverse events can assist in identifying adverse reactions, those adverse events where the treatment is considered to possibly be implicated in a causal role. The collection of adverse event and adverse reaction data is critically important in the assessment of drug safety. During a clinical trial, adverse events are collected systematically, but even in this context there is a possibility of under-reporting. In trials, collection of data on adverse events is a regulatory requirement. Outside of the context of a trial, reporting of adverse reactions after a treatment is available for usual clinical care (post-marketing) is generally considered to be poor. Identification of adverse events and their outcomes via electronic patient records could significantly enhance reporting of adverse events and reactions within trials in a longterm post-trial follow-up context and in post-marketing surveillance.

***D1. Reporting of adverse events or reactions within a trial or in post-trial longterm follow-up.***

**Scenario H;** Automated extraction of individual patient’s records containing adverse event data and associated clinical and prescribing data with transfer to the organisation conducting the clinical research, in the context of a trial with full consent for data access.

**Question 16**

Do you think that this scenario would be accepted by your institution (or an institution in your country if you are not based in a health care institution)?

(Tick one box)

|  | Yes |  |  | Don’t know |  |
| --- | --- | --- | --- | --- | --- |
|  | No |  |  | Not applicable |  |

**Question 17**

Do you think that this scenario would create ethical/ information governance concerns at your institution (or an institution in your country if you are not based in a health care institution)?

(Tick one box)

|  | Yes |  |  | Don’t know |  |
| --- | --- | --- | --- | --- | --- |
|  | No |  |  | Not applicable |  |

**Question 18**

Indicate your agreement/disagreement with the statement that 'Accumulating adverse event reports in this manner will significantly improve the reporting of adverse drug reactions during clinical trials'.

(Tick one box)

Strongly agree Agree Neither Disagree Strongly disagree

Provide any additional comments you might have on this topic:

***D2. Reporting of adverse events or reactions in a post-marketing context.***

**Scenario I;** If a clinician documents symptoms, findings or results that are suggestive of an Adverse Drug Reaction (ADR), then an automated extraction of the patient’s individual record could be used to help the clinician complete and submit an ADR reporting form to the marketing authority and the regulatory agency, as appropriate.

**Question 19**

Indicate whether you think that this scenario would require previous informed consent by patients for the use of their data.

(Tick one box)

|  | Yes |  |  | Don’t know |  |
| --- | --- | --- | --- | --- | --- |
|  | No |  |  | Not applicable |  |

**Question 20**

Do you think that that this scenario would be approved by your institution (or an institution in your country if you are not based in a health care institution)?

(Tick one box)

|  | Yes |  |  | Don’t know |  |
| --- | --- | --- | --- | --- | --- |
|  | No |  |  | Not applicable |  |

**Question 21**

Do you think that this scenario would create ethical/ information governance concerns at your institution (or an institution in your country if you are not based in a health care institution)?

(Tick one box)

|  | Yes |  |  | Don’t know |  |
| --- | --- | --- | --- | --- | --- |
|  | No |  |  | Not applicable |  |

**Question 22**

Indicate your agreement/disagreement with the statement that 'Accumulating adverse event reports in this manner will significantly improve the reporting of adverse drug reactions outside of the context of clinical trials'.

(Tick one box)

Strongly agree Agree Neither Disagree Strongly disagree

Provide any additional comments you might have on this topic:

**Scenario J;** In contrast to Scenario I, suppose that automated extraction of only periodic **aggregated summary** information (hospital and drug level) on adverse events was extracted from electronic records for reporting to the marketing authority and the regulatory agency, as appropriate.

**Question 23**

Indicate whether you think that this scenario would require previous informed consent by patients for the use of their data.

(Tick one box)

|  | Yes |  |  | Don’t know |  |
| --- | --- | --- | --- | --- | --- |
|  | No |  |  | Not applicable |  |

**Question 24**

Do you think that that this scenario would be approved by your institution (or an institution in your country if you are not based in a health care institution)?

(Tick one box)

|  | Yes |  |  | Don’t know |  |
| --- | --- | --- | --- | --- | --- |
|  | No |  |  | Not applicable |  |

**Question 25**

Do you think that data this scenario would create ethical/ information governance concerns at your institution (or an institution in your country if you are not based in a health care institution)?

(Tick one box)

|  | Yes |  |  | Don’t know |  |
| --- | --- | --- | --- | --- | --- |
|  | No |  |  | Not applicable |  |

**Question 26**

Indicate your agreement/disagreement with the statement that 'Accumulating adverse event reports in this manner will significantly improve the reporting of adverse drug reactions'.

(Tick one box)

Strongly agree Agree Neither Disagree Strongly disagree

Provide any additional comments you might have on this topic:

**Scenario K;** In the UK, the General Practitioners Research Database (GPRD), containing detailed de-identified longitudinal primary care data on hundreds of thousands of patients, is available for license by organisations conducting clinical research to assist in their study planning and for epidemiological studies. A similar system exists in France, known as the “PMSI” database.

**Question 27**

Do you think that out-licensing from your institution (or from an institution in your country if you are not based in a health care institution) of a large body of detailed pseudo-anonymised longitudinal secondary care (hospital) data to an organisation conducting research into post marketing drug safety would:

(Tick one box for each of a), b), c), and d))

a) Require prior patient level consent?

| Yes |  |  | Don’t know |  |
| --- | --- | --- | --- | --- |
| No |  |  | Not applicable |  |

b) Be likely to receive institutional approval?

| Yes |  |  | Don’t know |  |
| --- | --- | --- | --- | --- |
| No |  |  | Not applicable |  |

c) Raise significant ethical/information security concerns?

| Yes |  |  | Don’t know |  |
| --- | --- | --- | --- | --- |
| No |  |  | Not applicable |  |

d) Require Data Protection Authority or another regulatory external body approval?

| Yes |  |  | Don’t know |  |
| --- | --- | --- | --- | --- |
| No |  |  | Not applicable |  |

Provide any additional comments you might have on this topic:

**
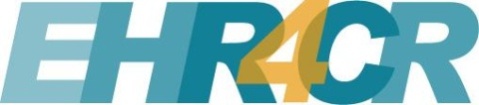
**

**Stakeholder interaction – Non - Ethics**

**Other Issues**

**(Questions 28-31)**

**
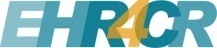
 Other aspects**

**Question 28**

Indicate your agreement/disagreement with the following as strong motivating factors for your institution’s participation in the EHR4CR platform (now or in the future). If you are not based in a healthcare institution, consider these factors for an institution in your country.

(Tick one box for each factor)

1. **Increased income generation from participation in more industry trials**

Strongly agree Agree Neither Disagree Strongly disagree

1. **Pressure from government or institution to participate in more pharma-industry studies**

Strongly agree Agree Neither Disagree Strongly disagree

1. **Providing patients with faster access to new generation medicines**

Strongly agree Agree Neither Disagree Strongly disagree

1. **Development of local Health Information Systems**

Strongly agree Agree Neither Disagree Strongly disagree

1. **Improvement of local data quality and health care**

Strongly agree Agree Neither Disagree Strongly disagree

1. **The potential to use EHR4CR platform to conduct academic studies**

Strongly agree Agree Neither Disagree Strongly disagree

1. **Opportunity to improve the quality of data in clinical trials**

Strongly agree Agree Neither Disagree Strongly disagree

1. **Opportunity to improve the efficiency of clinical trials**

Strongly agree Agree Neither Disagree Strongly disagree

Provide any additional motivating factors:

**Question 29**

Indicate your agreement/disagreement with the following as significant threats in your institution or country to the success of EHR4CR.

(Tick one box for each threat)

1. **Inadequate availability of key data fields in the patient record**

Strongly agree Agree Neither Disagree Strongly disagree

1. **Missing data in the patient record**

Strongly agree Agree Neither Disagree Strongly disagree

1. **Inadequacy of local Health Information Systems**

Strongly agree Agree Neither Disagree Strongly disagree

1. **Cost of upgrading local systems to be compatible with EHR4CR**

Strongly agree Agree Neither Disagree Strongly disagree

1. **Ethical committee concerns**

Strongly agree Agree Neither Disagree Strongly disagree

1. **Local Information Governance concerns**

Strongly agree Agree Neither Disagree Strongly disagree

1. **Data protection authorities**

Strongly agree Agree Neither Disagree Strongly disagree

1. **Concerns of Hospital management**

Strongly agree Agree Neither Disagree Strongly disagree

1. **Concerns of patients**

Strongly agree Agree Neither Disagree Strongly disagree

1. **Concerns of clinicians**

Strongly agree Agree Neither Disagree Strongly disagree

List any additional threats:

**Question 30;**

Indicate your agreement/disagreement with the statement 'I support the overall objectives of the EHR4CR project';

(Tick one box)

Strongly agree Agree Neither Disagree Strongly disagree

Provide any additional comments you might have on the EHR4CR project:

**Question 31;**

Would you be willing to be contacted again to answer follow-up questions?

| Yes |  |  |
| --- | --- | --- |
| No |  |  |

**
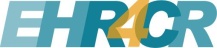
 Stakeholder interaction; Ethics**

EHR4CR Partner (interviewer)_______________________________ (*select from list*)

Interviewer Details:

| **Interviewer (*PRINT NAME*):** |  |
| --- | --- |
| **Interview Date:** |  |
| **Interviewer Email address:** |  |

By ticking this box you are giving us permission to store the above data for the purposes of reporting to the EHR4CR project team. All data will be subject to the Data Protection Act 1998

Interviewee category;

| - Ethics Committee representative |
| --- |

**
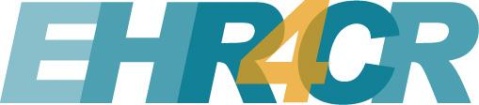
**

**Stakeholder interaction – Ethics**

**Project Areas A - D**

**(Questions 1 – 22)**

**
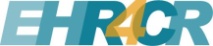
 Project Area A:**

**Using the EHR4CR Platform for Assessing clinical trial feasibility**

**Background**

In this context, access to patient data would be required to inform trial design and trial feasibility assessment. Therefore, ethical and information governance approval would be required for a generic process not necessarily associated with any particular trial.

*Informing study design:* The development of a trial design by an organisation involved in clinical research might require basic knowledge about the number of prevalent cases or the throughput of incident cases with a particular condition. Alternatively, there might be a need to know about the biological variability in a measurement of interest or in the rate of clinical outcomes of interest (e.g. death, re-admission etc).

*Informing feasibility:* Once a protocol has been outlined or even finalised, there will be a need to assess the number of potentially eligible patients at each clinical site to assess the feasibility of the study and to inform the required number of sites and the best sites to participate in the trial.

Infrastructure to answer the questions posed above can be established in different ways.

**Scenario A;** An electronic query would be sent to each participating centre that would be run against a local database established for clinical research purposes returning summary information only (e.g. counts and percentages). Data will be returned only if counts are sufficiently large to protect privacy. For example, information might be returned on the percentage of patients eligible for inclusion in a study.

**Scenario B;** An electronic query would be sent to each participating centre that would require the return of summary information cross-tabulated by a number of key inclusion/exclusion criteria. Data will be returned only if counts are sufficiently large to protect privacy. For instance, the number of eligible participants might be returned for combinations of gender (male/female) and diabetes status (not diabetic/type I/type II).

**Scenario C;** An electronic query would be sent to each participating centre that would require the return of pseudo-anonymised individual patient records containing patient level information on key inclusion/exclusion criteria and other variables of interest. On this occasion, the data would be collated at a trusted third party site, in Europe, so that the impact of variations in study design criteria could be explored. The records would not contain any patient identifiers; date of birth would be converted into age and recorded to nearest year.

**Scenario D;** An electronic query would be sent to each participating centre that would require the return of de-identified individual patient records containing patient level information on key inclusion/exclusion criteria. On this occasion, the data would be returned to the organisation conducting the clinical research that initiated the query, so that the impact of variations in study design criteria could be explored.

**Question 1**

For **each scenario**, indicate whether you think that data transfer would require previous informed consent by patients for the use of their data in this manner.

(Tick one box for each scenario)

| Scenario A | Yes |  |  | Don’t know |  |
| --- | --- | --- | --- | --- | --- |
|  | No |  |  | Not applicable |  |
|  |  |  |  |  |  |
| Scenario B | Yes |  |  | Don’t know |  |
|  | No |  |  | Not applicable |  |
|  |  |  |  |  |  |
| Scenario C | Yes |  |  | Don’t know |  |
|  | No |  |  | Not applicable |  |
|  |  |  |  |  |  |
| Scenario D | Yes |  |  | Don’t know |  |
|  | No |  |  | Not applicable |  |

Provide any additional comments you might have on this topic;

**Question 2**

For each scenario, indicate whether you think, in your opinion, that data transfer would be approved by an ethics committee in your country.

(Tick one box for each scenario)

| Scenario A | Yes |  |  | Don’t know |  |
| --- | --- | --- | --- | --- | --- |
|  | No |  |  | Not applicable |  |
|  |  |  |  |  |  |
| Scenario B | Yes |  |  | Don’t know |  |
|  | No |  |  | Not applicable |  |
|  |  |  |  |  |  |
| Scenario C | Yes |  |  | Don’t know |  |
|  | No |  |  | Not applicable |  |
|  |  |  |  |  |  |
| Scenario D | Yes |  |  | Don’t know |  |
|  | No |  |  | Not applicable |  |

Provide any additional comments you might have on this topic

**Question 3**

Indicate your agreement/disagreement with the statement that 'This approach to facilitating feasibility assessment would enhance the conduct of clinical trials'.

(Tick one box)

Strongly agree Agree Neither Disagree Strongly disagree

Provide any additional comments you might have on this topic

**
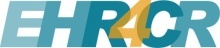
 Project Area B:**

**Use of the EHR4CR Platform for Facilitating Recruitment to Trials**

**Background**

**Scenario E;** Once a trial design has been finalised, all clinical trial approvals obtained and clinical investigators recruited and contracts completed, there is the opportunity to use routinely collected patient data to facilitate the identification of potentially eligible recruits for the trial. In this context the study inclusion/exclusion criteria would be provided in electronic form to the clinical site. An application would be run on behalf of the investigator against the local database to extract a list of potentially eligible patients for the local investigator to scrutinise, delete individuals from the list as appropriate based on local knowledge, and where appropriate generate letters of invitation to participate in the trial to the patients. No individual patient level data would be returned to the organisation conducting the clinical research prior to patient consent.

**Question 4a**

Indicate whether you think that this scenario would require previous informed consent by patients for the use of their data by the investigator/healthcare team.

(Tick one box)

|  | Yes |  |  | Don’t know |  |
| --- | --- | --- | --- | --- | --- |
|  | No |  |  | Not applicable |  |

**Question 4b**

Indicate whether you think that this scenario would require previous authorisation from Data Protection Authority or other external regulatory body.

(Tick one box)

|  | Yes |  |  | Don’t know |  |
| --- | --- | --- | --- | --- | --- |
|  | No |  |  | Not applicable |  |

**Question 5**

Do you think, in your opinion, that this scenario would be approved by an ethics committee in your country?

|  | Yes |  |  | Don’t know |  |
| --- | --- | --- | --- | --- | --- |
|  | No |  |  | Not applicable |  |

**Question 6**

Indicate your agreement/disagreement with the statement that 'This approach to facilitating patient recruitment would enhance the conduct of clinical trials'.

(Tick one box)

Strongly agree Agree Neither Disagree Strongly disagree

Provide any additional comments you might have on this topic:

**
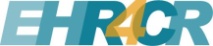
 Project Area C:**

**Use of the EHR4CR Platform for Facilitating Clinical Trial Conduct**

**Background**

In this context, it is assumed that the patient has been recruited into the trial and provided full informed consent for the extraction of data from their electronic record and for addition of new information into the patient record.

**Scenario F;** Extraction of data from the patient record: Accurate collection of clinical trial data can be time consuming, often requiring manual transcribing of information already held in patient routinely collected health records and resolution of queries caused by inaccurate data entry. Examples of data that might be available in electronic records are, demographics, medical history, prescription information, routine measurements (e.g. Blood Pressure, Body Mass Index, pulse). The ability to upload high quality data automatically into the trial database could facilitate trial conduct. There would be an option to allow the investigator to approve each data transfer.

**Question 7**

Do you think that this scenario would be approved by an ethics committee in your country?

(Tick one box)

|  | Yes |  |  | Don’t know |  |
| --- | --- | --- | --- | --- | --- |
|  | No |  |  | Not applicable |  |

**Question 8**

Indicate your agreement/disagreement with the statement that 'This approach to facilitating trial conduct would enhance the quality of clinical trials.'.

(Tick one box)

Strongly agree Agree Neither Disagree Strongly disagree

Provide any additional comments you might have on this topic:

**Scenario G;** Return of trial specific data to the patient’s electronic record: Data collected for trial purposes that supplement what would be collected in clinical practice are seldom returned to the patient’s record. The ability to automatically enter such information into the patient’s record could in theory enhance future patient care. However, some have raised concerns that data collected out of the usual clinical context, using non-standard techniques and possibly including measurements with unproven clinical relevance, could cause confusion and might not always be helpful for clinical management. Such information could arise from more frequently recorded measurements, specialised measurements on the patient or from images, ECGs, laboratory or tissue samples, PRO, QoL data etc.

**Question 9**

Do you think that this scenario would be approved by an ethics committee in your country?

(Tick one box)

|  | Yes |  |  | Don’t know |  |
| --- | --- | --- | --- | --- | --- |
|  | No |  |  | Not applicable |  |

**Question 10**

Indicate your agreement/disagreement with the statement that 'This approach to facilitating trial conduct would enhance the quality of clinical trials.'

(Tick one box)

Strongly agree Agree Neither Disagree Strongly disagree

**Question 11**

Do you think that this scenario could create concerns that the additional information might be misunderstood by other physicians treating the patient due to unfamiliar measurements or measurements obtained by unfamiliar methods?

(Tick one box)

|  | Yes |  |  | Don’t know |  |
| --- | --- | --- | --- | --- | --- |
|  | No |  |  | Not applicable |  |

**Question 12**

Do you think that this scenario would create fewer concerns if the additional information was separated from the usual patient record?

(Tick one box)

|  | Yes |  |  | Don’t know |  |
| --- | --- | --- | --- | --- | --- |
|  | No |  |  | Not applicable |  |

Provide any additional comments you might have on this topic, including potential benefits of this approach:

**
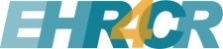
 Project Area D;**

**Use of the EHR4CR platform for Adverse Event Reporting**

**Background**

An adverse event is a change to a patient’s health occurring after initiation of a treatment. Collection of data on adverse events can assist in identifying adverse reactions, those adverse events where the treatment is considered to possibly be implicated in a causal role. The collection of adverse event and adverse reaction data is critically important in the assessment of drug safety. During a clinical trial, adverse events are collected systematically, but even in this context there is a possibility of under-reporting. In trials, collection of data on adverse events is a regulatory requirement. Outside of the context of a trial, reporting of adverse reactions after a treatment is available for usual clinical care (post-marketing) is generally considered to be poor. Identification of adverse events and their outcomes via electronic patient records could significantly enhance reporting of adverse events and reactions within trials in a longterm post-trial follow-up context and in post-marketing surveillance.

***D1. Reporting of adverse events or reactions within a trial or in post-trial longterm follow-up.***

**Scenario H;** Automated extraction of individual patient’s records containing adverse event data and associated clinical and prescribing data with transfer to the organisation conducting the clinical research, in the context of a trial with full consent for data access.

**Question 13**;

Do you think that this scenario would be approved by an ethics committee in your country?

(Tick one box)

|  | Yes |  |  | Don’t know |  |
| --- | --- | --- | --- | --- | --- |
|  | No |  |  | Not applicable |  |

**Question 14;**

Indicate your agreement/disagreement with the statement that 'This approach to facilitating adverse event reporting would enhance the evaluation of the safety of medicines'.

(Tick one box)

Strongly agree Agree Neither Disagree Strongly disagree

Provide any additional comments you might have on this topic:

***D2. Reporting of adverse events or reactions in a post-marketing context.***

**Scenario I;** If a clinician documents symptoms, findings or results that are suggestive of an Adverse Drug Reaction (ADR), then an automated extraction of the patient’s individual record could be used to help the clinician complete and submit an ADR reporting form to the marketing authority and the regulatory agency, as appropriate.

**Question 15**

Indicate whether you think that this scenario would require previous informed consent by patients for the use of their data.

(Tick one box)

|  | Yes |  |  | Don’t know |  |
| --- | --- | --- | --- | --- | --- |
|  | No |  |  | Not applicable |  |

**Question 16**

Do you think that that this scenario would be approved by an ethics committee in your country?

(Tick one box)

|  | Yes |  |  | Don’t know |  |
| --- | --- | --- | --- | --- | --- |
|  | No |  |  | Not applicable |  |

**Question 17**

Indicate your agreement/disagreement with the statement that 'This approach to facilitating adverse event reporting would enhance the evaluation of the safety of medicines'.

(Tick one box)

Strongly agree Agree Neither Disagree Strongly disagree

Provide any additional comments you might have on this topic:

**Scenario J;** In contrast to Scenario I, suppose that automated extraction of only periodic **aggregated summary** information (hospital and drug level) on adverse events was extracted from electronic records for reporting to the marketing authority and the regulatory agency, as appropriate.

**Question 18**

Indicate whether you think that this scenario would require previous informed consent by patients for the use of their data.

(Tick one box)

|  | Yes |  |  | Don’t know |  |
| --- | --- | --- | --- | --- | --- |
|  | No |  |  | Not applicable |  |

**Question 19**

Do you think that that this scenario would be approved by your institution (or an institution in your country if you are not based in a health care institution)?

(Tick one box)

|  | Yes |  |  | Don’t know |  |
| --- | --- | --- | --- | --- | --- |
|  | No |  |  | Not applicable |  |

**Question 20**

Do you think that data this scenario would create ethical/ information governance concerns at your institution (or an institution in your country if you are not based in a health care institution)?

(Tick one box)

|  | Yes |  |  | Don’t know |  |
| --- | --- | --- | --- | --- | --- |
|  | No |  |  | Not applicable |  |

**Question 21**

Indicate your agreement/disagreement with the statement that 'Accumulating adverse event reports in this manner will significantly improve the reporting of adverse drug reactions'.

(Tick one box)

Strongly agree Agree Neither Disagree Strongly disagree

Provide any additional comments you might have on this topic:

**Scenario K;** In the UK, the General Practitioners Research Database (GPRD), containing detailed de-identified longitudinal primary care data on hundreds of thousands of patients, is available for license by organisations conducting clinical research to assist in their study planning and for epidemiological studies. A similar system exists in France, known as the “PMSI” database.

**Question 22**

Do you think that out-licensing from your institution (or from an institution in your country if you are not based in a health care institution) of a large body of detailed pseudo-anonymised longitudinal secondary care (hospital) data to an organisation conducting research into post marketing drug safety would:

(Tick one box for each of a), b), c), and d))

a) Require prior patient level consent?

| Yes |  |  | Don’t know |  |
| --- | --- | --- | --- | --- |
| No |  |  | Not applicable |  |

b) Be likely to receive institutional approval?

| Yes |  |  | Don’t know |  |
| --- | --- | --- | --- | --- |
| No |  |  | Not applicable |  |

c) Raise significant ethical/information security concerns?

| Yes |  |  | Don’t know |  |
| --- | --- | --- | --- | --- |
| No |  |  | Not applicable |  |

d) Require Data Protection Authority or another regulatory external body approval?

| Yes |  |  | Don’t know |  |
| --- | --- | --- | --- | --- |
| No |  |  | Not applicable |  |

Provide any additional comments you might have on this topic:

**
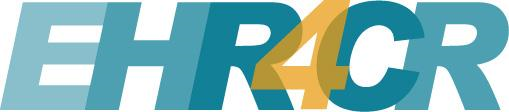
**

**Stakeholder interaction – Ethics**

**Other aspects (Q23 – 26)**

**
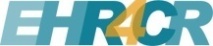
 Other aspects**

**Question 23**

Indicate your agreement/disagreement with the following as strong motivating factors for your institution’s participation in the EHR4CR platform (now or in the future). If you are not based in a healthcare institution, consider these factors for an institution in your country.

(Tick one box for each factor)

1. **Increased income generation from participation in more industry trials**

Strongly agree Agree Neither Disagree Strongly disagree

1. **Pressure from government or institution to participate in more pharma-industry studies**

Strongly agree Agree Neither Disagree Strongly disagree

1. **Providing patients with faster access to new generation medicines**

Strongly agree Agree Neither Disagree Strongly disagree

1. **Development of local Health Information Systems**

Strongly agree Agree Neither Disagree Strongly disagree

1. **Improvement of local data quality and health care**

Strongly agree Agree Neither Disagree Strongly disagree

1. **The potential to use EHR4CR platform to conduct academic studies**

Strongly agree Agree Neither Disagree Strongly disagree

1. **Opportunity to improve the quality of data in clinical trials**

Strongly agree Agree Neither Disagree Strongly disagree

1. **Opportunity to improve the efficiency of clinical trials**

Strongly agree Agree Neither Disagree Strongly disagree

Provide any additional motivating factors:

**Question 24;**

Indicate your agreement/disagreement with the following as significant threats in your institution or country to the success of EHR4CR.

(Tick one box for each threat)

1. **Inadequate availability of key data fields in the patient record**

Strongly agree Agree Neither Disagree Strongly disagree

1. **Missing data in the patient record**

Strongly agree Agree Neither Disagree Strongly disagree

1. **Inadequacy of local Health Information Systems**

Strongly agree Agree Neither Disagree Strongly disagree

1. **Cost of upgrading local systems to be compatible with EHR4CR**

Strongly agree Agree Neither Disagree Strongly disagree

1. **Ethical committee concerns**

Strongly agree Agree Neither Disagree Strongly disagree

1. **Local Information Governance concerns**

Strongly agree Agree Neither Disagree Strongly disagree

1. **Data protection authorities**

Strongly agree Agree Neither Disagree Strongly disagree

1. **Concerns of Hospital management**

Strongly agree Agree Neither Disagree Strongly disagree

1. **Concerns of patients**

Strongly agree Agree Neither Disagree Strongly disagree

1. **Concerns of clinicians**

Strongly agree Agree Neither Disagree Strongly disagree

List any additional threats:

**Question 25;**

Indicate your agreement/disagreement with the statement 'I support the overall objectives of the EHR4CR project';

(Tick one box)

Strongly agree Agree Neither Disagree Strongly disagree

Provide any additional comments you might have on the EHR4CR project:

**Question 26;**

Would you be willing to be contacted again to answer follow-up questions?

| Yes |  |  |
| --- | --- | --- |
| No |  |  |
